# Handball participation and bone mineral accrual during growth: A 1‐year longitudinal controlled study in young male athletes

**DOI:** 10.14814/phy2.71133

**Published:** 2026-10-13

**Authors:** Kawther Missawi, Maha Gasmi, Antonella Muscella, Tahir Volkan Aslan, Özgür Eken, Monira I. Aldhahi, Mohamed Zouch

**Affiliations:** ^1^ Laboratory of Exercise Physiology and Pathophysiology: From Integrated to Molecular “Biology, Medicine and Health”, UR12ES06, Faculty of Medicine Ibn Jazar Sousse University of Sousse Sousse Tunisia; ^2^ Higher Institute of Sport and Physical Education of Ksar Said Manouba University Tunis Tunisia; ^3^ Department of Biological and Environmental Science and Technologies (DiSTeBA) University of Salento Lecce Italy; ^4^ Department of Travel, Tourism and Entertainment Services, Erdemli Vocational School Mersin University Mersin Türkiye; ^5^ Department of Physical Education and Sport Teaching Inonu University Malatya Türkiye; ^6^ Department of Rehabilitation Sciences, College of Health and Rehabilitation Sciences Princess Nourah Bint Abdulrahman University Riyadh Saudi Arabia; ^7^ Department of Sport Sciences and Physical Activity, College of Education University of Ha'il Hail Saudi Arabia

**Keywords:** bone area, bone health, bone mineral content, bone mineral density, dual‐energy X‐ray absorptiometry (DXA), handball, longitudinal study, physical activity, skeletal development

## Abstract

Multidirectional sports such as handball may provide beneficial mechanical stimuli, but longitudinal evidence in male children remains limited. This study examined one‐year differences in bone mineral density (BMD), bone mineral content (BMC), and bone area (BA) between male children engaged in regular handball practice and age‐matched controls. This non‐randomized longitudinal study included 72 healthy male children aged 10–12 years: handball group (HG, *n* = 36) and control group (CG, *n* = 36). Whole‐body and regional bone outcomes were assessed using dual‐energy X‐ray absorptiometry. Between‐group differences at follow‐up were evaluated using ANCOVA, while longitudinal changes were examined using linear mixed‐effects models. Handball players exhibited greater adjusted BMD at follow‐up than controls, with the largest effect at the lumbar spine (adjusted mean difference = 0.034 g/cm^2^; 95% CI: 0.012–0.056; *η* ^*2*^ *p* = 0.14). Higher BMD was also observed at the whole body, total hip, lower limbs, femoral neck, and selected upper‐limb sites. Adjusted BMC was greater at the lumbar spine, total hip, lower limbs, and selected radial regions. Increases in lean mass were positively associated with BMD gains at the whole body, hip, and femoral neck, whereas associations with BMC were more localized and those with BA were limited. These findings suggest that handball participation is associated with greater skeletal mineral accrual during growth.

## 1 INTRODUCTION

Bone development during childhood and adolescence represents a critical determinant of lifelong skeletal health. Growth is accompanied by substantial increases in body size and mechanical demands, requiring progressive accumulation of bone mass to maintain skeletal strength and structural integrity (Chevalley & Rizzoli, 2022). Bone is a dynamic tissue that undergoes continuous adaptation, and parameters such as bone mineral density (BMD), bone mineral content (BMC), and bone area (BA) are commonly used to characterize skeletal development (Wang et al., 2024). The acquisition of these skeletal properties during growth is influenced by multiple factors, including genetics, nutrition, and physical activity (Del'Arco et al., 2021; Inzaghi et al., 2022).

Adequate nutrition and regular participation in physical activity are essential contributors to optimal bone mineralization and peak bone mass acquisition (Barr & McKay, 1998; Proia et al., 2021). In particular, mechanical loading generated by high‐impact and weight‐bearing activities stimulates skeletal adaptation during periods of rapid growth. Previous studies have shown that children and adolescents participating in high‐impact sports exhibit greater BMD, BMC, and bone structural characteristics compared with peers engaged in lower‐impact activities (Costa et al., 2022; Dias Quiterio et al., 2011). Studies focusing specifically on prepubertal populations have similarly reported greater bone mass in young athletes than in non‐athletic peers. For example, prepubertal tennis players showed greater bone mass at several skeletal sites compared with non‐athletic controls, although the adaptations were particularly evident in the dominant upper limb (Sanchis‐Moysi et al., 2010). Similarly, prepubescent males participating in volleyball or basketball exhibited greater bone mass at several skeletal sites compared with controls, supporting the beneficial influence of sport participation on skeletal development before puberty (Zouch et al., 2016). These findings highlight childhood and early adolescence as important windows for promoting skeletal development and potentially reducing the risk of osteoporosis later in life (Gunter et al., 2012).

Among youth sports, handball represents a potentially osteogenic activity due to its combination of multidirectional movements, intermittent high‐intensity actions, and repeated mechanical loading. Training involves frequent jumping, landing, sprinting, rapid changes of direction, and throwing actions, which expose both weight‐bearing and upper‐limb skeletal sites to sport‐specific mechanical stimuli. Previous studies have suggested that handball participation is associated with favorable adaptations in bone health and physical fitness in young populations (Hornstrup et al., 2019; Kovács et al., 2024; Martone et al., 2024).

Compared with other youth sports, handball may provide a distinctive mechanical stimulus to the skeleton because it combines high‐impact, multidirectional, and sport‐specific loading of both the lower and upper limbs. The combination of repetitive lower‐limb impact loading and upper‐limb loading associated with throwing may therefore contribute to site‐specific skeletal adaptations across multiple anatomical regions. Consistent with this premise, previous studies have reported greater BMD and/or BMC at several skeletal sites in handball players compared with non‐athletic controls (Boshnjaku et al., 2016; Krahenbühl et al., 2019; Vicente‐Rodriguez et al., 2004). Studies in adolescent female athletes have further reported greater bone mass in handball players than in soccer players at the upper limbs, lower limbs, trunk, ribs, spine, and total body, suggesting that the skeletal effects of handball may extend beyond predominantly weight‐bearing sites (Fagundes et al., 2022). In addition, Ubago‐Guisado et al. (2015), in a study of 200 girls participating in different sports, reported higher intertrochanteric BMD in prepubertal handball players compared with controls. Among pubertal girls, both handball and soccer players showed higher total BMD and BMD at the arms, pelvis, femoral neck, intertrochanteric region, and Ward's triangle compared with controls (Ubago‐Guisado et al., 2015).

Importantly, comparisons between different sports suggest that the skeletal response may not be uniform across athletic activities. In prepubertal boys, volleyball and basketball were both associated with greater bone mass compared with controls, but differences in the anatomical distribution of bone adaptations were also observed between the two sports (Zouch et al., 2016). Likewise, among prepubertal and peripubertal female footballers, greater bone mass was observed compared with non‐athletic controls, particularly at weight‐bearing skeletal sites (Plaza‐Carmona et al., 2016). Together, these findings suggest that, although participation in osteogenic sports is generally associated with greater skeletal development during the prepubertal period, the magnitude and anatomical distribution of these adaptations may depend on the specific mechanical demands of each sport.

Although numerous studies and systematic reviews have investigated the effects of sports participation on bone health and have reported differences in bone properties between physically active and less active or non‐athletic individuals (Milanović et al., 2022), evidence specifically addressing skeletal adaptations to handball during the prepubertal period remains limited. In particular, previous studies of handball players have predominantly focused on adolescents or adults, whereas longitudinal evidence focusing specifically on prepubescent athletes is scarce. Furthermore, the relative contribution of changes in bone mineralization (BMD and BMC) compared with changes in bone size (BA) during growth remains insufficiently explored. Given the distinctive combination of lower‐ and upper‐limb mechanical loading inherent to handball, it is therefore plausible that handball may induce a different anatomical pattern of skeletal adaptation compared with sports characterized predominantly by lower‐limb weight‐bearing activity. However, whether handball results in greater or qualitatively different skeletal adaptations than other sports during the prepubertal period remains unclear.

Against this background, the present study addresses an important gap by providing a longitudinal one‐year comparison of prepubescent male participants engaged in regular handball practice and age‐matched non‐athletic controls. In addition to assessing whole‐body and regional BMD, the study simultaneously evaluates BMC and BA, allowing changes in bone mineralization to be considered alongside changes in bone size during growth. Furthermore, both weight‐bearing and non‐weight‐bearing skeletal sites are examined, enabling a more comprehensive assessment of the site‐specific skeletal adaptations potentially associated with the combined lower‐ and upper‐limb loading characteristics of handball. Therefore, the aim of the present study was to investigate one‐year differences in BMD, BMC, and BA between male children engaged in regular handball practice and age‐matched controls, including both weight‐bearing and non‐weight‐bearing skeletal sites.

## 2 MATERIALS AND METHODS

### 2.1 Study design

This was a one‐year longitudinal, non‐randomized study designed to investigate differences in skeletal development between prepubescent male children regularly engaged in handball practice and age‐matched controls.

Participants were recruited through collaborations with several schools in the Sahel region of Tunisia, involving physical education teachers and school health departments. The handball participants were recruited from different schools and were affiliated with different handball teams, whereas the control participants did not participate in organized handball training. Despite being affiliated with different teams, all handball players followed the same standardized training program throughout the study period. Male children were categorized into either a handball group (HG) or a control group (CG) according to their habitual physical activity participation.

Before study enrollment, participants and their parents or legal guardians received detailed information regarding the study procedures, potential risks and benefits, and their rights as research participants. They were explicitly informed that participation was entirely voluntary and that both the participants and their parents or legal guardians had the right to refuse participation or withdraw consent at any time, without providing a reason and without any consequences. Written informed consent was obtained from the parents or legal guardians, and written assent was obtained from the participants before their inclusion in the study. All procedures were approved by the Ethics Committee of Farhat Hached Hospital, Sousse, Tunisia (LR19ES10), and were conducted in accordance with the Declaration of Helsinki.

Assessments were performed at baseline (T0) and after 1 year of follow‐up (T1). Measurements included anthropometric characteristics, body composition, and skeletal parameters assessed by dual‐energy X‐ray absorptiometry (DXA), dietary calcium intake, physical activity level, and physical fitness performance tests, including the 20‐m shuttle run and jump tests. All assessments were conducted at the Laboratory of Evaluation of Human Biological Performance, Farhat Hached Hospital.

### 2.2 Participants

An a priori power analysis was conducted using G*Power software (version 3.1.9.7; Heinrich Heine University Düsseldorf, Germany). The calculation was based on a repeated‐measures ANOVA (within–between interaction) with two groups and two measurement occasions (baseline and 12‐month follow‐up). Whole‐body bone mineral density (BMD) was selected as the primary outcome for sample size estimation. Assuming a medium effect size (Cohen's *f* = 0.25), a significance level of *α* = 0.05, a statistical power (1 − *β*) of 0.80, and a moderate correlation between repeated measurements (*r* = 0.60), the minimum required sample size was estimated to be 30 participants.

A total of 72 male children completed the 12‐month follow‐up and were included in the final analyses: 36 in the handball group (HG; age: 10.88 ± 0.92 years; body mass: 41.75 ± 11.53 kg; height: 151.0 ± 5.6 cm) and 36 in the control group (CG; age: 10.61 ± 0.57 years; body mass: 34.86 ± 5.96 kg; height: 144.0 ± 4.7 cm) (Figure 1).

**FIGURE 1:**
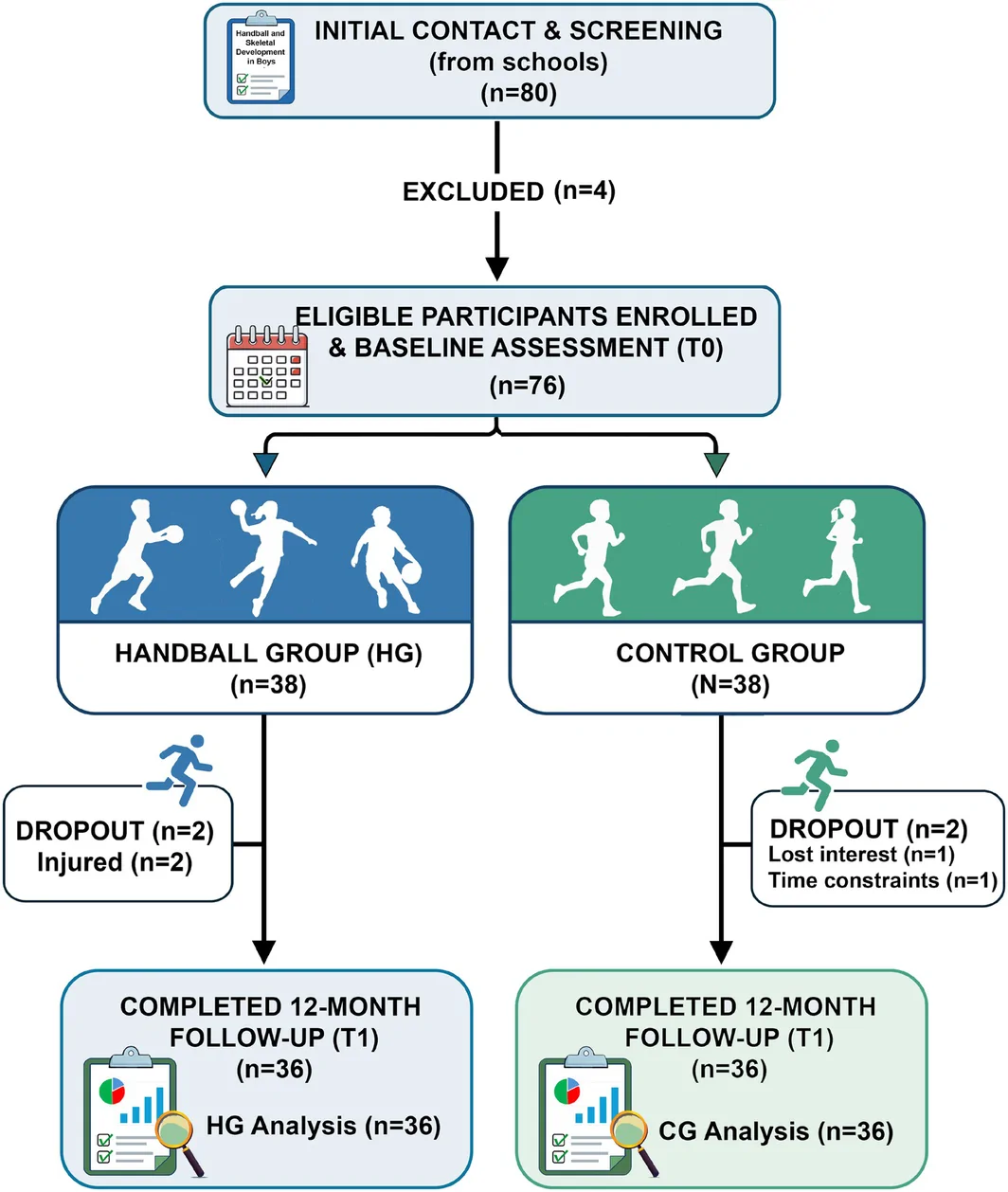
Participant flow diagram. CG, control group; HG, handball group.

Participants in the handball group had been engaged in organized handball training for at least 2 years before study enrolment and continued their regular training throughout the study period. Participants in the control group had not participated in organized sports or structured physical activity outside the standard school physical education curriculum during the previous 2 years or throughout the follow‐up period. Thus, the control group continued their usual school physical education activities but did not undertake additional organized or structured physical activity during the study.

The inclusion criteria were: (i) male sex; (ii) age between 10 and 12 years; (iii) Tanner Stage I or II at baseline; (iv) regular participation in handball training (≥3 sessions per week) for at least 2 years for the HG, or no organized sport participation for at least 2 years for the CG; and (v) completion of all scheduled assessments during the 12‐month follow‐up. The exclusion criteria included: (i) chronic metabolic, endocrine, or musculoskeletal disorders affecting bone metabolism (e.g., diabetes mellitus, thyroid disorders, or growth hormone therapy); (ii) fractures, major musculoskeletal injuries, or orthopedic surgery within the previous 12 months; (iii) use of medications or nutritional supplements known to influence bone metabolism (including systemic corticosteroids, vitamin D, or calcium supplementation) during the previous 6 months; and (iv) adherence to restrictive dietary patterns (e.g., vegetarian, vegan, or ketogenic diets) that could substantially affect nutrient intake relevant to bone health.

Pubertal maturation was assessed by a pediatrician trained in standardized Tanner staging procedures. In male participants, genital development and pubic hair were evaluated according to the Tanner criteria (Tanner & Whitehouse, 1976), ranging from Stage I (prepubertal) to Stage V (full pubertal maturation). At baseline, participants were predominantly classified as Tanner Stage I, with two participants in the Control Group classified as Stage II. When the stages of genital development and pubic hair did not correspond, the final Tanner stage was determined by the assessing pediatrician based on the overall clinical assessment of pubertal development. Pubertal status was reassessed at the end of the 12‐month follow‐up to document pubertal progression.

To further account for growth‐related influences on bone outcomes, statistical analyses were adjusted for height growth velocity.

### 2.3 Measurement of body composition and physical performance

Anthropometric measurements were obtained for all participants following standardized procedures, with participants wearing light clothing and barefoot. Height was measured to the nearest 0.001 m using a wall‐mounted stadiometer (model S‐220; Seca, Hanover, MD, USA), and body mass was recorded to the nearest 0.1 kg using an electronic scale (model 770; Seca). Body mass index (BMI) was calculated as body mass divided by height squared (kg/m^2^).

Body composition was assessed using dual‐energy X‐ray absorptiometry (DXA; Lunar Prodigy, GE Healthcare, Madison, WI, USA; software version 3.6). Total fat mass and lean mass were obtained from whole‐body DXA scans performed at the Rheumatology Department of Sousse University Hospital.

Height growth velocity was calculated as the change in standing height between baseline (T0) and follow‐up (T1), divided by the time elapsed between the two measurements, and expressed in cm/year, according to the following formula:

$$\text{Height velocity}\;\left(\mathrm{cm}/\text{year}\right)=\left[\text{height}\;\mathrm{at}\;\text{T1}\;\left(\mathrm{cm}\right)-\text{height}\;\mathrm{at}\;\text{T0}\;\left(\mathrm{cm}\right)\right]/\text{time elapsed between measurements}\;\left(\text{years}\right).$$

Cardiorespiratory fitness was assessed using the Léger 20‐m shuttle run test, and performance was recorded as the total distance completed (m). Habitual physical activity level outside organized handball training was assessed using Bratteby's questionnaire (Bratteby et al., 1997).

Lower‐limb explosive performance was evaluated through jump tests, including the horizontal jump and squat jump tests.

#### 2.3.1 Dietary assessment

Dietary intake was assessed at baseline using a three‐day dietary record, including two weekdays and one weekend day (Table 1). Participants recorded all foods and beverages consumed during the assessment period. The dietary records were analyzed using Bilnut SCDA Nutrisoft software (version 2.01, 1990; Cerelles, France) to estimate average daily energy and nutrient intake. In addition to calcium intake, the analysis provided estimates of average daily energy, protein, carbohydrate, fat, vitamin D, and calcium intake.

**TABLE 1:** Baseline dietary intake in the control and handball groups. Dietary variables included daily energy, protein, carbohydrate, fat, calcium, and vitamin D intake.

Dietary variable	Control group	Handball group	*p*
Energy (kcal/day)	2366 ± 254	2367 ± 256	0.95
Protein (g/day)	73.92 ± 8.27	74.03 ± 11.07	0.77
Carbohydrate (g/day)	348.72 ± 48.71	349.64 ± 38.77	0.64
Fat (g/day)	79.08 ± 8.30	79.94 ± 7.90	0.71
Vitamin D (μg/day)	9.78 ± 1.82	10.14 ± 1.39	0.532

*Note*: Data are presented as mean ± standard deviation. *p*‐values were calculated using Welch's *t*‐test for normally distributed variables and the Mann–Whitney *U* test for non‐normally distributed variables. A two‐sided *p*‐value <0.05 was considered statistically significant.

### 2.4 Bone mass measurements

Bone parameters, including bone mineral density (BMD, g/cm^2^), bone mineral content (BMC, g), and bone area (BA, cm^2^), were assessed using dual‐energy X‐ray absorptiometry (DXA; Lunar Prodigy, GE Healthcare, Madison, WI, USA; software version 3.6). All measurements were performed at the Rheumatology Department of Sousse University Hospital by the same trained technician experienced in densitometry procedures.

The DXA system was calibrated daily using the manufacturer‐provided phantom before scanning sessions. The same device and software were used for all assessments throughout the study to ensure measurement consistency. Whole‐body scans were performed to obtain total BMD, BMC, and BA, while regional analyses were conducted for specific skeletal sites, including the lumbar spine, femoral neck, total hip, lower limbs, upper limbs, and radius regions.

Participants were scanned in the supine position according to standardized manufacturer procedures, with limbs positioned consistently to minimize variability. Metallic objects were removed before scanning, and all scans were reviewed for quality control and potential motion artifacts. When necessary, scans were repeated. DXA analyses were performed using the enCORE software (version 3.6).

Bone parameters were analyzed as absolute values and as percentage changes from baseline to follow‐up to evaluate longitudinal differences over the one‐year study period.

### 2.5 Handball training program

The handball participants belonged to different handball teams recruited from the participating schools in the Sahel region of Tunisia. Although the participants were affiliated with different teams and trained under different coaches, all teams followed the same standardized training program throughout the study period. The program was identical across teams in terms of training content, structure, frequency, and duration. Throughout the study period, one investigator from the research team monitored adherence to the standardized program by maintaining regular contact with the coaches and verifying the implementation of the prescribed training content and schedule. Any deviations from the standardized program were recorded. This monitoring procedure was implemented to ensure comparable training exposure among all participants in the handball group.

The handball training program was conducted under the supervision of certified coaches and followed a structured training plan throughout the one‐year study period. Training sessions were performed three times per week on an indoor court, with each session lasting approximately 90 min.

Each session consisted of a standardized structure including warm‐up, technical and tactical training, game‐based activities, and cool‐down. The warm‐up phase (15–20 min) included dynamic movements, mobility exercises, and low‐intensity ball‐handling activities. The technical and tactical component (15–25 min) focused on fundamental handball skills, including passing, dribbling, shooting, defensive actions, and positional play. Training also incorporated sprinting, acceleration, agility drills, and small‐sided games to reproduce the intermittent and multidirectional demands of handball.

The main training component (20–30 min) consisted of match‐based exercises emphasizing offensive and defensive transitions, teamwork, and tactical decision‐making. Training intensity and complexity were progressively adapted according to players' development and performance level. Each session ended with a cool‐down period including low‐intensity recovery activities and stretching.

### 2.6 Statistical analysis

All statistical analyses were performed using IBM SPSS Statistics (version 25.0; IBM Corp., Armonk, NY, USA). Data are presented as mean ± standard deviation (SD), and statistical significance was set at *p* < 0.05.

Prior to statistical analyses, the normality and distributional characteristics of all continuous variables, including anthropometric, dietary, body composition, and DXA‐derived outcomes (BMD, BMC, and BA), were assessed using the Shapiro–Wilk test and examination of skewness. Statistical methods were selected according to the distributional characteristics of the variables.

Differences in anthropometric variables, body composition parameters (including regional lean and fat mass), and dietary calcium intake were analyzed using linear mixed‐effects models, with Group (handball vs. Control) and Time (baseline vs. Follow‐up) included as fixed factors and participant included as a random intercept to account for within‐subject correlation across repeated measurements. Estimated marginal means and post hoc contrasts were examined where appropriate.

To examine between‐group differences at follow‐up, analysis of covariance (ANCOVA) was performed for bone mineral density (BMD), bone mineral content (BMC), and bone area (BA) at all skeletal regions. Group (handball vs. Control) was included as the fixed factor, and models were adjusted for baseline values of the respective outcome variables and height growth velocity. Additional covariates included height, lean mass, and dietary calcium intake to control for potential confounding factors influencing bone development.

Potential outliers in the DXA‐derived outcomes were examined, particularly observations showing large percentage changes from baseline to follow‐up. Individual observations exceeding 3 SD from the mean were reviewed for potential measurement or data‐entry errors. No measurement or data‐entry errors were identified, and these observations were therefore retained in the primary analyses. Sensitivity analyses were additionally performed after excluding the identified outliers using the same ANCOVA models as in the primary analyses; the results and interpretation of the between‐group comparisons remained unchanged.

Changes in whole‐body and regional BMD, BMC, and BA over time were further analyzed using linear mixed‐effects models (LMMs). The models included the interaction Group × Time as fixed effects, with participant included as a random intercept to account for within‐subject correlation across repeated measurements. Covariates included height, growth velocity, lean mass, and dietary calcium intake to account for individual differences in musculoskeletal development during growth. Effect sizes are reported as partial eta squared (*η* ^*2*^ *p*).

When significant Group × Time interactions were detected, pairwise comparisons with Bonferroni adjustment were performed to control for type I error. To account for multiple comparisons across skeletal regions, false discovery rate (FDR) correction using the Benjamini–Hochberg procedure was applied.

The skeletal regions analyzed represent pre‐specified anatomical sites commonly examined in pediatric DXA studies, and the analyses were primarily focused on clinically relevant outcomes.

For between‐group comparisons of baseline dietary variables, Welch's *t*‐test was used for normally distributed variables, whereas the Mann–Whitney *U* test was used for non‐normally distributed variables.

## 3 RESULTS

### 3.1 Participant characteristics, anthropometry, and physical fitness outcomes

Participants' age, anthropometric parameters, physical fitness, daily calcium intake, and physical activity level are summarized in (Table 2).

**TABLE 2:** Age, anthropometric data, calcium intake, and physical fitness in handball and control groups valued before and after intervention.

	T0	T1	T0	T1
	Control group *n* = 36	Control group *n* = 36	Handball group *n* = 36	Handball group *n* = 36
Age (year)	10.61 ± 0.57	11.58 ± 0.59	10.88 ± 0.92	11.77 ± 0.91
Weight (kg)	34.86 ± 5.96	38.94 ± 6.79	**41.75 ± 11.53** ^a^ **	**44.88 ± 12.47** ^b^ **
Height (cm)	144 ± 4.7	149 ± 5.1	151 ± 5.6	155 ± 6.4
BMI (kg/m^2^)	17.12 ± 2.75	17.39 ± 3.14	**18.73 ± 3.34** ^a^ **	**19.07 ± 3.79** ^b^ *
Lean mass (kg)	26.34 ± 3.08	29.05 ± 3.39	27.18 ± 6.57	**32.29 ± 5.99** ^b^ **
Fat mass (kg)	6.28 ± 4.96	9.10 ± 5.50	7.97 ± 6.91	10.62 ± 7.45
Calcium intake (mg/d)	760.00 ± 101.1	807.72 ± 197.0	771.22 ± 122.0	817.08 ± 187.9
Physical activity level	38.00 ± 1.38	36.78 ± 7.61	38.44 ± 1.24	38.44 ± 1.26
20mSRT (distance) (m)	527.86 ± 65.85	517.86 ± 65.85	825.48 ± 71.93^a^, ***	884.72 ± 71.93^b^, ***
SJ (cm)	19.38 ± 3.65	20.05 ± 3.47	**32.22 ± 7.05** ^a^ ***	**35.13 ± 7.08** ^b^ ***
HJ (cm)	136.22 ± 20.82	141. ± 23.65	**156.38 ± 12.55** ^a^ ***	**168.97 ± 12.24** ^b^ ***
Tanner stage	Stage I: *n* = 34 (94%), Stage II: *n* = 2 (6%)	Stage II: *n* = 29 (81%), Stage I: *n* = 7 (19%)	Stage I *n* = 36 (100%)	Stage II *n* = 31 (86%), Stage I: *n* = 5 (14%)

*Note*: Bold values indicate statistical significance.

Abbreviations: 20mSRT, 20‐metre shuttle run test; BMI, body mass index; CG, control group; HG, Handball group; HJ, horizontal jump; SJ, squat jump; T0, before intervention; T1, after intervention; VO_2_max, maximum oxygen consumption.

^a^ Significant difference between handball group (HG) and control group (CG) before intervention (T0).

^b^ Significant difference between handball group (HG) and control group (CG) after intervention (T1).

* *p* < 0.05.

** *p* < 0.001.

*** *p* < 0.0001.

Linear mixed‐effects models revealed significant main effects of group and time for height (group: *F*(1,105) = 46.96, *p* < 0.001, *η* ^2^ *p* = 0.309; time: *F*(1,105) = 30.82, *p* < 0.001, *η* ^2^ *p* = 0.227) and body mass (group: *F*(1,104.61) = 12.82, *p* < 0.001, *η* ^2^ *p* = 0.109; time: *F*(1,104.39) = 6.24, *p* = 0.014, *η* ^2^ *p* = 0.056), indicating that participants in both groups grew over time and that the handball group remained taller and heavier throughout the study. However, no significant group × time interactions were observed for either variable (height: *β* = 0.005, 95% CI −0.009 to 0.019; body mass: *β* = 0.42, 95% CI −0.35 to 1.18), suggesting similar longitudinal trajectories between groups.

No significant main effects or interaction were observed for BMI, and the group × time interaction was not significant (*F*(1,70) = 2.04, *p* = 0.157; *β* = 0.29, 95% CI −0.11 to 0.69).

For total lean mass, significant main effects of group (*F*(1,43.35) = 5.99, *p* = 0.018, *η* ^2^ *p* = 0.121) and time (*F*(1,70) = 66.45, *p* < 0.001, *η* ^2^ *p* = 0.655) were observed, whereas the group × time interaction was not significant (*F*(1,70) = 1.53, *p* = 0.225; *β* = −402.84, 95% CI −1040.90 to 235.22). Likewise, total fat mass demonstrated significant main effects of group (*F*(1,37.28) = 5.59, *p* = 0.023, *η* ^*2*^ *p* = 0.130) and time (*F*(1,64.47) = 6.77, *p* = 0.011, *η* ^*2*^ *p* = 0.095), with no significant interaction (*β* = 81.07, 95% CI −458.42 to 620.56).

Daily calcium intake remained stable throughout the study, with no significant effects of group, time, or their interaction (group × time: *F*(1,70) = 0.45, *p* = 0.503; *β* = −8.96, 95% CI −35.06 to 17.14).

Complete results of the linear mixed‐effects models, including F statistics, degrees of freedom, *p*‐values, effect sizes, and confidence intervals, are presented in Table S1.

### 3.2 Bone mineral density analysis

Baseline BMD values are presented in Table 3. Before the intervention (T0), the handball group exhibited significantly higher BMD only at the left arm compared with the control group. Following the intervention (T1), ANCOVA adjusted for baseline values, height growth velocity, height, lean mass, and dietary calcium intake revealed significantly higher BMD in the handball group across several skeletal regions.

**TABLE 3:** Comparison of bone mineral density (BMD) in handball and control groups valued before and after intervention.

BMD (g/cm^2^)	T0	T1	T0	T1
	Control group	Control group	Handball group	Handball group
Whole body	0.932 ± 0.040	0.939 ± 0.043	0.952 ± 0.070	**0.985 ± 0.070** ^b^ *
Lumbar spine	0.729 ± 0.059	0.772 ± 0.062	0.760 ± 0.081	**0.829 ± 0.0824** ^b^ **
*Weight‐bearing sites*				
Left leg 0,016	0.94 ± 0.061	0.990 ± 0.063	1.009 ± 0.136^a^, *	**1.079 ± 0.139** ^b^ *
Right leg	0.93 ± 0.061	0.992 ± 0.074	0.978 ± 0.195	**1.070 ± 0.124** ^b^ **
Left femoral neck	0.844 ± 0.045	0.865 ± 0.043	0.883 ± 0.116	**0.941 ± 0.097** ^b^ ***
Right femoral neck	0.867 ± 0.075	0.912 ± 0.088	0.912 ± 0.100	0.949 ± 0.093
Left total hip	0.875 ± 0.034	0.900 ± 0.043	0.918 ± 0.114^a^, *	**0.966 ± 0.105** ^b^ **
Right total hip	0.889 ± 0.073	0.920 ± 0.073	0.912 ± 0.107	**0.970 ± 0.104** ^b^ **
*Non‐Weight‐bearing sites*				
Left arm	0.662 ± 0.037	0.693 ± 0.047	0.703 ± 0.053^a^, **	**0.741 ± 0.061** ^b^ *
Right arm	0.674 ± 0.035	0.717 ± 0.133	0.725 ± 0.070^a^, **	0.770 ± 0.071
Left ultradistal radius	0.267 ± 0.028	0.290 ± 0.034	0.286 ± 0.047	0.306 ± 0.050
Right ultradistal radius	0.267 ± 0.027	0.288 ± 0.031	0.284 ± 0.061	**0.319 ± 0.064** ^b^ *
Left 1/3 radius	0.472 ± 0.048	0.500 ± 0.050	0.487 ± 0.056	0.515 ± 0.063
Right 1/3 radius	0.475 ± 0.043	0.504 ± 0.044	0.490 ± 0.061	0.526 ± 0.058
Left whole radius	0.375 ± 0.034	0.399 ± 0.036	0.385 ± 0.040	0.413 ± 0.043
Right whole radius	0.377 ± 0.032	0.397 ± 0.033	0.390 ± 0.052	**0.420 ± 0.051** ^b^ *

*Note*: Bold values indicate statistical significance.

^a^ Handball group vs control group before intervention (T0).

^b^ Handball group vs control group after intervention (T1).

* *p* < 0.05.

** *p* < 0.001.

*** *p* < 0.0001.

Whole‐body and lumbar spine BMD were significantly greater in the handball group at follow‐up. In addition, adjusted percentage gains in BMD were significantly higher than those of the control group at both the whole body (3.8 ± 4.09% vs. 2.2 ± 5.12%) and lumbar spine (9.0 ± 8.53% vs. 6.11 ± 5.37%) (both *p* < 0.05; Figure 2).

**FIGURE 2:**
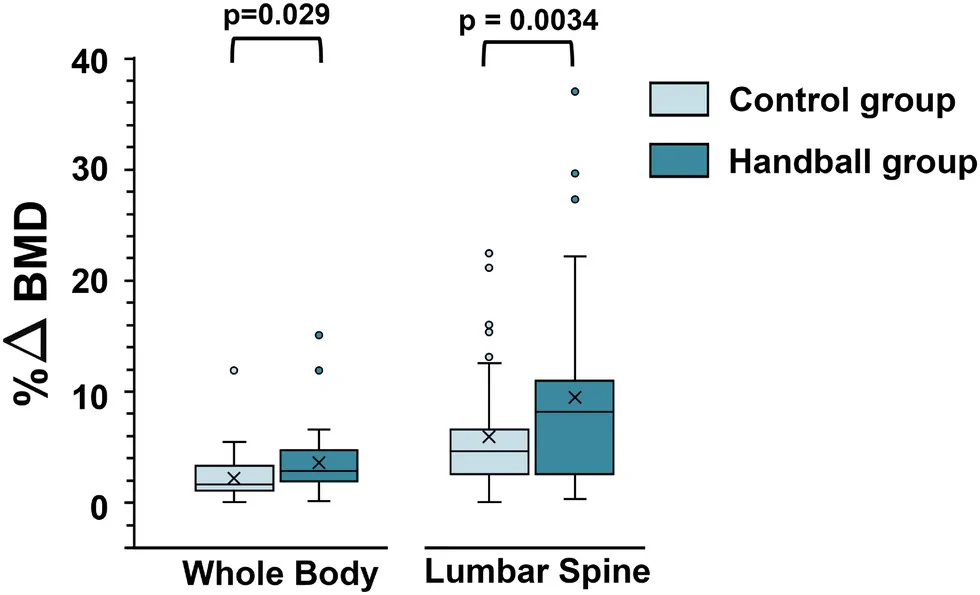
Percentage changes in bone mineral density (%ΔBMD). Box‐and‐whisker plots illustrating the percentage change in bone mineral density (%ΔBMD) from baseline (T0) to follow‐up (T1) for the whole body and lumbar spine in the handball group and the control group. Boxes represent the interquartile range (IQR; 25th–75th percentiles), the horizontal line indicates the median, the cross (×) denotes the mean, whiskers extend to 1.5 × IQR, and circles represent individual outliers. Between‐group differences were assessed using ANCOVA‐adjusted comparisons; exact *p*‐values are reported on the graphs.

At weight‐bearing sites, the handball group demonstrated significantly greater BMD at the left and right legs, left femoral neck, and both total hip regions compared with controls (all *p* < 0.05; Table 2, Figure 3a). Among non‐weight‐bearing sites, significantly higher BMD was observed at the left arm, right ultradistal radius, and right whole radius, whereas no significant between‐group differences were detected at the remaining upper‐limb sites (Table 3, Figure 3b).

**FIGURE 3:**
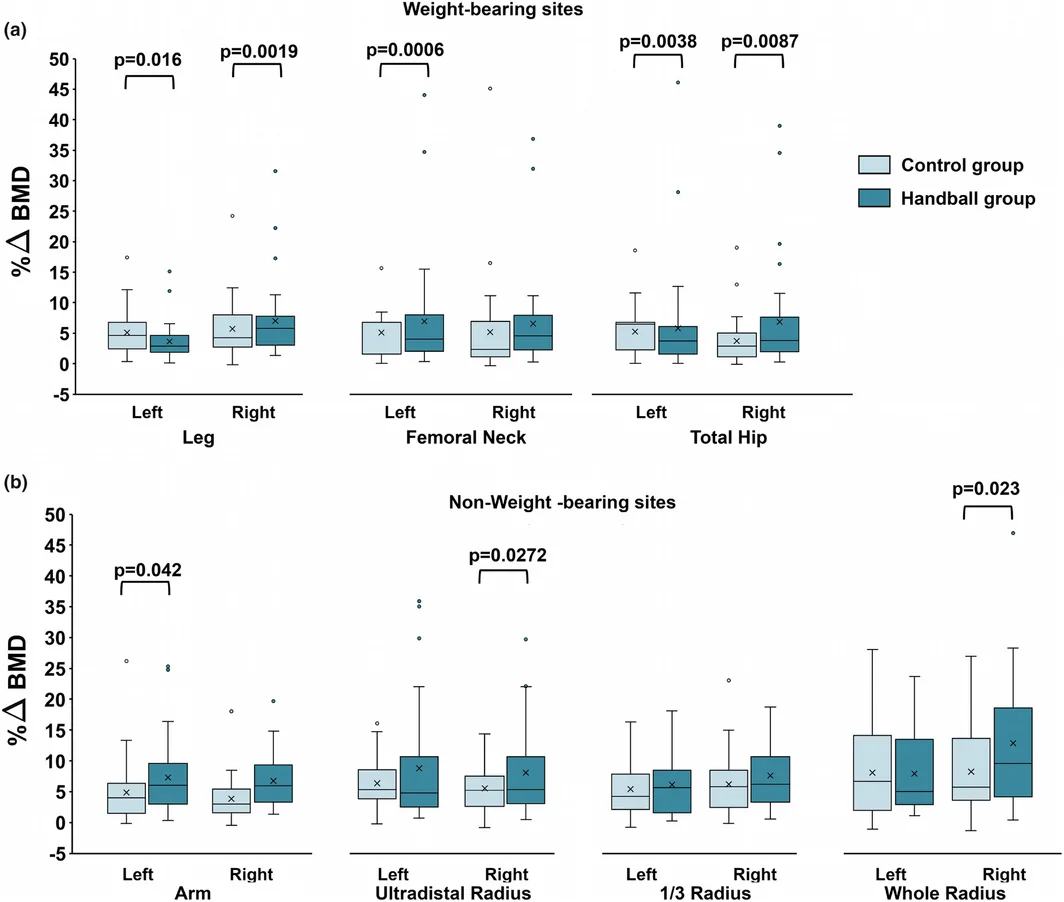
Percentage changes in bone mineral density (%ΔBMD) across weight‐bearing sites (a) and non‐weight‐bearing sites (b). Box‐and‐whisker plots illustrating the percentage change in bone mineral density (%ΔBMD) from baseline (T0) to follow‐up (T1) for general anatomical sites in the handball group and the control group. Boxes represent the interquartile range (IQR; 25th–75th percentiles), the horizontal line indicates the median, the cross (×) denotes the mean, whiskers extend to 1.5 × IQR, and circles represent individual outliers. Between‐group differences were assessed using ANCOVA‐adjusted comparisons; exact *p*‐values are reported on the graph.

Finally, linear mixed‐effects models evaluating group × time interactions revealed significant differential changes between handball players and controls across specific skeletal regions, including the left leg (*F*(1, 138) = 4.660, *p* = 0.031, FDR *p* = 0.091), lumbar spine (*F*(1, 138) = 5.866, *p* = 0.015, FDR *p* = 0.091), left femoral neck (*F*(1, 138) = 8.325, *p* = 0.004, FDR *p* = 0.066), and whole body (*F*(1, 138) = 5.728, *p* = 0.017, FDR *p* = 0.091). Complete interaction models for all skeletal regions are detailed in Table S2.

Overall, these findings indicate that regular handball participation during growth was associated with greater bone mineral accrual, particularly at clinically relevant weight‐bearing skeletal sites, supporting the potential osteogenic benefits of impact‐loading exercise during growth.

### 3.3 Bone mineral content analysis

Baseline BMC values were significantly higher in the handball group at several skeletal sites, including the whole body, lumbar spine, femoral neck, total hip, upper limbs, and lower limbs (Table 3). To account for these baseline differences, follow‐up comparisons were performed using ANCOVA adjusted for baseline values, height growth velocity, height, lean mass, and dietary calcium intake.

At follow‐up, the adjusted ANCOVA revealed significantly greater BMC in the handball group at the lumbar spine, left leg, left and right total hip, and right one‐third radius compared with controls (all *p* < 0.05; Table 4).

**TABLE 4:** Comparison of bone mineral content (BMC) in handball and control group before and after intervention.

BMC (g)	T0	T1	T0	T1
	Control group	Control group	Handball group	Handball group
Whole body	1321.14 ± 39.92	1461.94 ± 192.27	1524.05 ± 375.60^a^, **	1710.13 ± 391.16
Lumbar spine	20.66 ± 3.19	23.10 ± 3.59	22.94 ± 4.48^a^, *	**26.63 ± 5.08** ^b^ *
*Weight‐bearing sites*				
Left leg	241.93 ± 37.50	275.88 ± 45.38	302.90 ± 95.75^a^, ***	**350.97 ± 98.19** ^b^ *
Right leg	240.90 ± 39.92	277.22 ± 50.76	305.25 ± 88.88^a^, ***	351.20 ± 91.21
Left femoral neck	1.72 ± 0.37	2.80 ± 0.55	2.19 ± 0.86^a^, **	3.17 ± 0.82
	2.17 ± 0.59	2.91 ± 0.77	2.22 ± 0.72	3.21 ± 0.85
Left total hip	18.20 ± 1.35	20.33 ± 1.69	21.71 ± 1.5086^a^, **	**24.51 ± 1.54** ^b^ *
Right total hip	18.61 ± 3.41	20.91 ± 2.51	**21.96 ± 4.30** ^a^ **	**25.03 ± 4.92** ^b^ *
*Non‐weight‐bearing sites*				
Left arm	62.68 ± 13.68	73.15 ± 13.99	80.66 ± 23.98^a^, ***	93.37 ± 26.64
Right arm	60.74 ± 11.29	70.98 ± 11.72	81.12 ± 25.63^a^, ***	95.54 ± 30.16
Left ultradistal radius	0.73 ± 0.09	0.82 ± 0.10	0.80 ± 0.17^a^, *	0.93 ± 0.23
Right ultradistal radius	0.72 ± 0.09	0.823 ± 0.10	0.82 ± 0.22^a^, *	0.974 ± 0.25
Left 1/3 radius	1.07 ± 0.12	1.17 ± 0.13	1.13 ± 0.19	1.24 ± 0.19
Right 1/3 radius	1.09 ± 0.11	1.17 ± 0.11	1.15 ± 0.19	**1.27 ± 0.18** ^b^ *
Left whole radius	3.77 ± 0.51	4.24 ± 0.53	4.21 ± 0.93^a^, *	4.85 ± 1.05
Right whole radius	3.74 ± 0.43	4.25 ± 0.48	4.33 ± 1.05^a^, *	4.99 ± 1.07

*Note*: Bold values indicate statistical significance.

^a^ Handball group vs control group before intervention (T0).

^b^ Handball group vs control group after intervention (T1).

* *p* < 0.05.

** *p* < 0.001.

*** *p* < 0.0001.

Handball players also exhibited significantly greater percentage gains in whole‐body BMC (13.07 ± 10.33% vs. 10.65 ± 6.46%) and lumbar spine BMC (16.91 ± 13.42% vs. 12.11 ± 8.87%) than controls (both *p* < 0.05; Figure 4).

**FIGURE 4:**
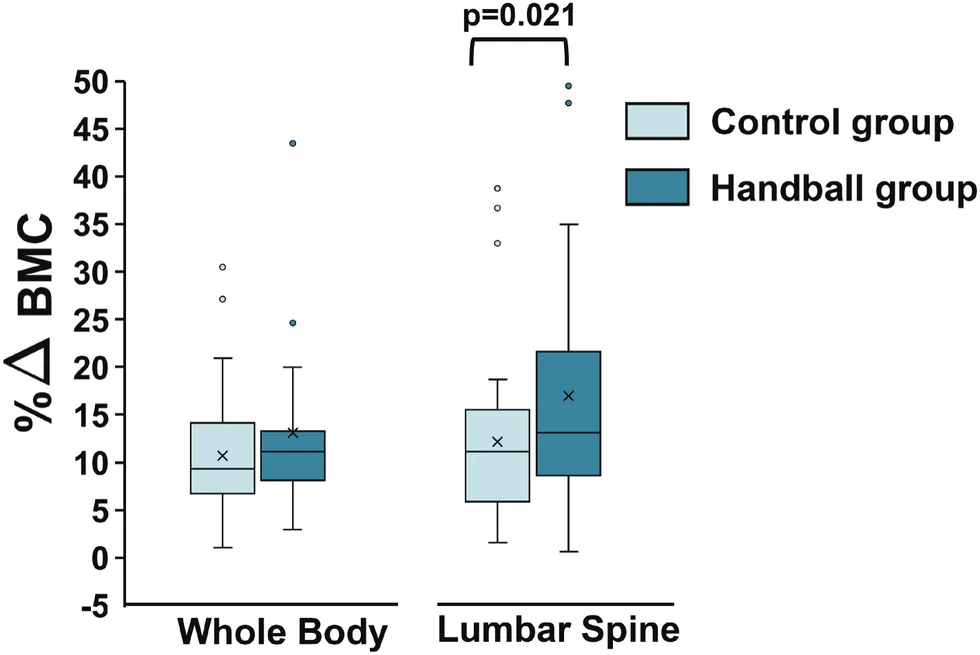
Percentage changes in bone mineral content (%ΔBMC). Box‐and‐whisker plots illustrating the percentage change in bone mineral density (%ΔBMC) from baseline (T0) to follow‐up (T1) for the whole body and lumbar spine in the handball group and the control group. Boxes represent the interquartile range (IQR; 25th–75th percentiles), the horizontal line indicates the median, the cross (×) denotes the mean, whiskers extend to 1.5 × IQR, and circles represent individual outliers. Between‐group differences were assessed using ANCOVA‐adjusted comparisons; exact *p*‐values are reported on the graph.

At T1, the adjusted ANCOVA model revealed significant group effects for several skeletal sites. Among weight‐bearing regions, handball players showed greater BMC at the left leg (*p* < 0.05), left total hip (*p* < 0.05), and right total hip (*p* < 0.05) compared with controls after adjustment for baseline BMC, height growth velocity, height, lean mass, and dietary calcium intake. Among non‐weight‐bearing regions, a significant adjusted group effect was observed only at the right one‐third radius (*p* < 0.05). Overall, the between‐group differences were more consistently observed at weight‐bearing sites, particularly in the lower limbs and hip, although some regional differences were also present at non‐weight‐bearing sites (Figure 5).

**FIGURE 5:**
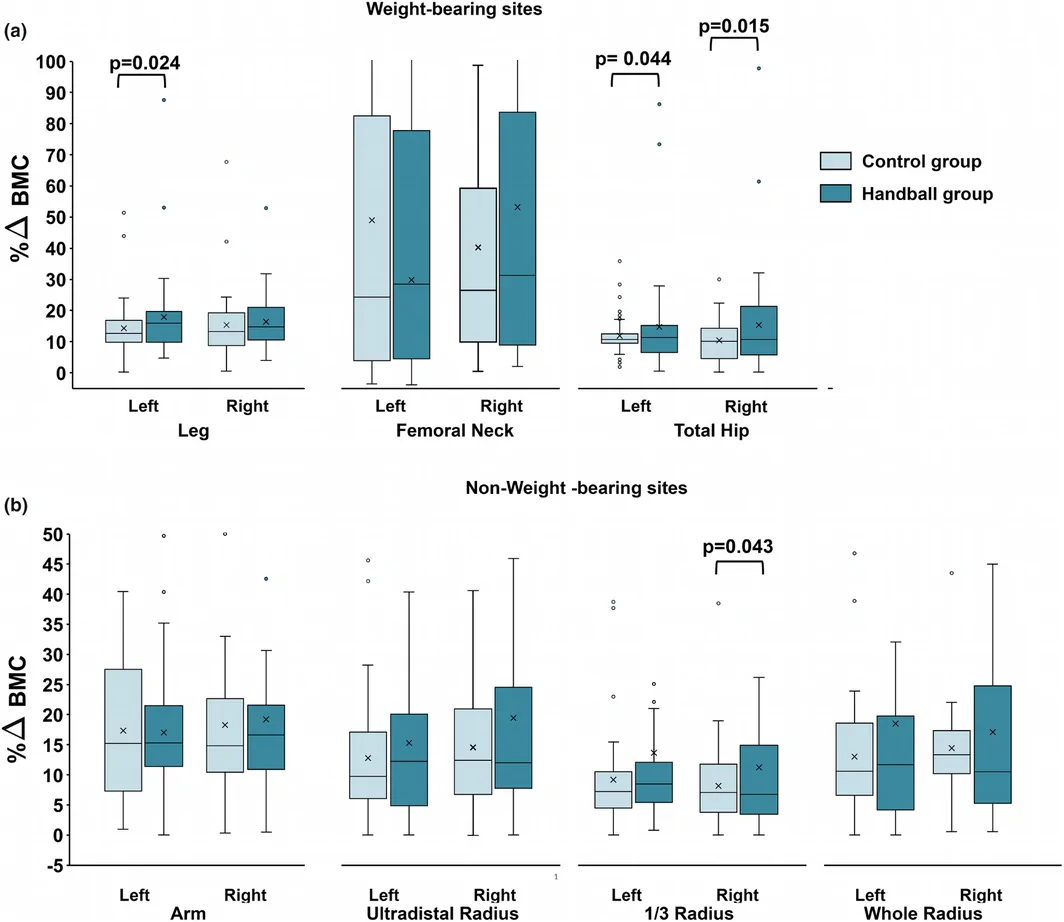
Percentage changes in bone mineral content (%ΔBMC) across weight‐bearing sites (a) and non‐weight‐bearing sites (b). Box‐and‐whisker plots illustrating the percentage change in bone mineral density (%ΔBMC) from baseline (T0) to follow‐up (T1) for general anatomical sites in the handball group and the control group. Boxes represent the interquartile range (IQR; 25th–75th percentiles), the horizontal line indicates the median, the cross (×) denotes the mean, whiskers extend to 1.5 × IQR, and circles represent individual outliers. Between‐group differences were assessed using ANCOVA‐adjusted comparisons; exact *p*‐values are reported on the graph.

Finally, linear mixed‐effects models evaluating group × interactions revealed significant differential changes between handball players and controls across specific skeletal regions, including the left leg (*F*(1, 138) = 6.366, *p* = 0.012, FDR *p* = 0.137), right arm (*F*(1, 138) = 4.621, *p* = 0.032, FDR *p* = 0.179), and lumbar spine (*F*(1, 138) = 5.790, *p* = 0.016, FDR *p* = 0.137). Complete interaction models for all skeletal regions are detailed in Table S3.

Overall, these findings indicate that regular handball participation during growth was associated with greater bone mineral accrual, particularly at clinically relevant weight‐bearing skeletal sites, supporting the potential osteogenic benefits of impact‐loading exercise during growth.

### 3.4 Bone area analysis

Baseline BA values differed between groups at several skeletal sites (Table 5). Follow‐up comparisons were therefore performed using ANCOVA adjusted for baseline values, height growth velocity, height, lean mass, and dietary calcium intake.

**TABLE 5:** Comparison of bone area (BA) in the handball and control groups before and after intervention.

BA (cm^2^)	T0	T1	T0	T1
	Control group	Control group	Handball group	Handball group
Whole body	1415.22 ± 131.86	1536.77 ± 167.00	1587.61 ± 289.93	1726.55 ± 281.49
Lumbar spine	27.56 ± 3.08	30.66 ± 3.55	29.64 ± 3.14	32.28 ± 3.84
*Weight‐bearing sites*				
Left leg	254.33 ± 25.45	278.85 ± 30.45	294.38 ± 56.89	321.25 ± 52.85
Right leg	254.66 ± 28.76	278.60 ± 33.08	294.88 ± 57.87	323.27 ± 56.75
Left femoral neck	2.08 ± 0.47	3.30 ± 0.62	2.40 ± 0.87	3.37 ± 0.99
Right femoral neck	2.42 ± 0.63	3.35 ± 0.77	2.44 ± 0.72	3.45 ± 0.88
Left total hip	20.57 ± 1.10	22.65 ± 1.54	23.27 ± 3.95	25.35 ± 3.80
Right total hip	20.60 ± 3.36	23.28 ± 2.43	23.86 ± 3.71	25.88 ± 3.65
*Non‐weight‐bearing sites*				
Left arm	93.47 ± 16.60	107.33 ± 16.18	111.91 ± 25.64	**126.91 ± 26.07** ^b^ *
Right arm	90.63 ± 13.35	106.26 ± 25.40	108.77 ± 26.32	**124.38 ± 28.42** ^b^ *
Left ultradistal radius	2.69 ± 0.29	2.90 ± 0.23	2.77 ± 0.37	**3.05 ± 0.33** ^b^ *
Right ultradistal radius	2.66 ± 0.29	2.93 ± 0.31	2.84 ± 0.32	3.07 ± 0.29
Left 1/3 radius	2.24 ± 0.19	2.39 ± 0.19	2.29 ± 0.29	2.45 ± 0.21
Right 1/3 radius	2.25 ± 0.18	2.40 ± 0.20	2.31 ± 0.25	2.47 ± 0.22
Left whole radius	9.95 ± 1.05	10.82 ± 1.00	10.68 ± 1.36	**11.85 ± 1.56** ^b^ *
Right whole radius	9.84 ± 1.02	10.87 ± 1.10	10.90 ± 1.65	11.93 ± 1.60

*Note*: Bold values indicate statistical significance.

^b^ Handball group vs control group after intervention (T1).

* *p* < 0.05.

Although whole‐body and lumbar spine BA increased in both groups over time, no significant between‐group differences were observed at follow‐up after adjustment for baseline values and covariates (both *p* > 0.05) (Figure 6).

**FIGURE 6:**
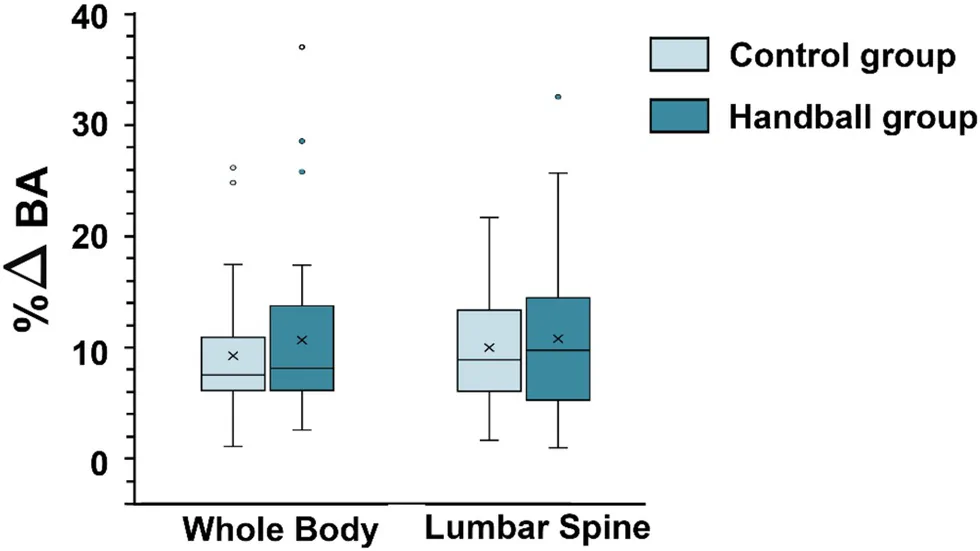
Percentage changes in bone area (%ΔBA). Box‐and‐whisker plots illustrating the percentage change in bone mineral density (%ΔBA) from baseline (T0) to follow‐up (T1) for the whole body, and lumbar spine in the handball group and the control group. Boxes represent the interquartile range (IQR; 25th–75th percentiles), the horizontal line indicates the median, the cross (×) denotes the mean, whiskers extend to 1.5 × IQR, and circles represent individual outliers.

At the regional level, the handball group exhibited higher adjusted BA at selected non‐weight‐bearing sites, including the left arm, right arm, left ultradistal radius, and left whole radius (all nominal *p* < 0.05; Table 4, Figure 7).

**FIGURE 7:**
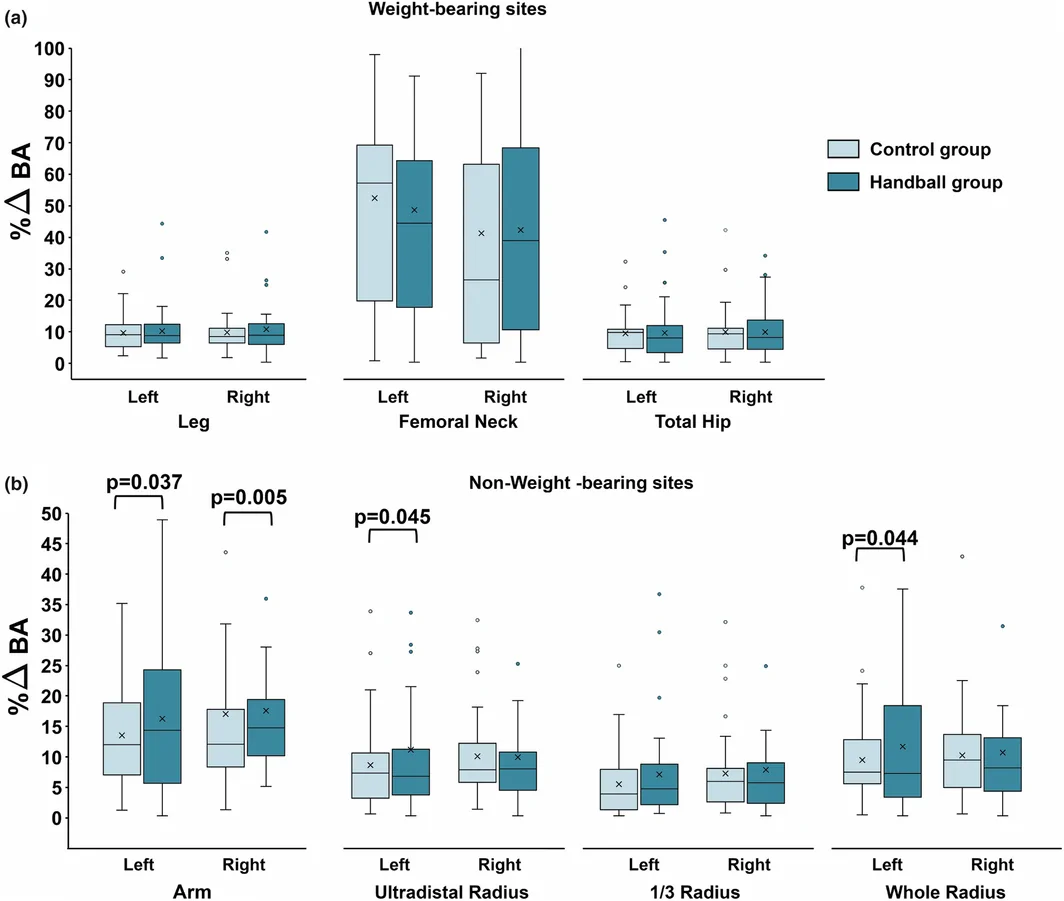
Percentage changes in bone area (%ΔBA) across weight‐bearing sites (a) and non‐weight‐bearing sites (b). Box‐and‐whisker plots illustrating the percentage change in bone mineral density (%ΔBA) from baseline (T0) to follow‐up (T1) for general anatomical sites in the handball group and the control group. Boxes represent the interquartile range (IQR; 25th–75th percentiles), the horizontal line indicates the median, the cross (×) denotes the mean, whiskers extend to 1.5 × IQR, and circles represent individual outliers. Between‐group differences were assessed using ANCOVA‐adjusted comparisons; exact *p*‐values are reported on the graph.

Linear mixed‐effects models identified nominal group × time interactions for several skeletal regions, including the whole body, lumbar spine, left leg, left femoral neck, and both total hip sites. However, none of these interactions remained statistically significant after false discovery rate (FDR) correction (all *p*FDR > 0.05; Table S4), indicating comparable longitudinal changes in bone area between the two groups.

Overall, these findings suggest that regular handball participation was associated with relatively limited between‐group differences in bone area, whereas greater between‐group differences were observed in bone mineral accrual and bone mineral density.

### 3.5 Relationship between lean mass changes and bone accrual

To investigate the contribution of lean mass accretion to skeletal development, linear regression analyses were performed using changes in bone parameters (ΔBMD, ΔBMC, and ΔBA; follow‐up minus baseline) as dependent variables and Δlean mass as the independent variable, adjusting for age, sex, height, and dietary calcium intake.

Greater increases in lean mass were significantly associated with greater gains in BMD at the whole body, bilateral total hip, and femoral neck sites (*p* < 0.05; Table 6). For BMC, significant associations with Δlean mass were observed at the right total hip and right one‐third radius, while the association at the right arm was borderline (*p* = 0.050). In contrast, associations between lean mass accretion and BA were generally weak, with significant relationships observed only at the right and left whole radius (*p* = 0.026 and *p* = 0.006, respectively).

**TABLE 6:** Linear regression analysis examining the association between changes in lean mass (Δlean mass) and gains in bone parameters (ΔBMD, ΔBMC, and ΔBA), adjusted for age, sex, height, and dietary calcium intake.

Skeletal site	Outcome	*β* (unstandardized)	95% CI	*p*
Whole body	ΔBMD	0.0001	0.0000–0.0002	0.008
Left femoral neck	ΔBMD	0.0001	0.0000–0.0002	0.031
Right femoral neck	ΔBMD	0.0001	0.0000–0.0002	0.024
Left total hip	ΔBMD	0.0001	0.0000–0.0002	0.012
Right total hip	ΔBMD	0.0001	0.0000–0.0002	0.015
Right total hip	ΔBMC	0.0003	0.0001–0.0005	0.005
Right Us radius	ΔBMC	<0.0001	0.0000–0.0001	0.011
Left whole radius	ΔBA	0.0001	0.0000–0.0001	0.006
Right whole radius	ΔBA	0.0001	0.0000–0.0001	0.026

Overall, these findings indicate that greater lean mass accretion was primarily associated with increased bone mineralization, particularly BMD at weight‐bearing skeletal sites, whereas associations with bone area were limited to specific forearm regions.

## 4 DISCUSSION

The present study investigated the effects of regular handball participation on skeletal development during growth, with particular emphasis on bone mineral density (BMD), bone mineral content (BMC), bone area (BA), and the contribution of lean mass changes to skeletal adaptation. Overall, our findings indicate that handball participation was associated with greater skeletal mineral accrual, particularly at clinically relevant weight‐bearing sites, whereas differences in bone size were less evident. Furthermore, increases in lean mass were positively associated with gains in BMD and, to a lesser extent, BMC, providing additional insight into the potential mechanisms linking muscular development and skeletal adaptation during growth.

Regular handball participation was associated with superior physical fitness, as trained participants demonstrated higher VO_2_max, squat jump, and horizontal jump performance compared with controls. VO_2_max is a widely accepted marker of cardiorespiratory fitness, and higher values are consistently observed in physically trained compared with non‐trained individuals (Srivastava et al., 2024). In addition to cardiovascular adaptations, the specific demands of handball—including repeated jumping, sprinting, rapid changes of direction, and frequent accelerations and decelerations—provide substantial mechanical stimuli to the developing skeleton. Previous intervention studies have demonstrated that high‐impact activities such as jumping can increase bone mineral accrual in children, particularly at the total body and lower‐limb regions (Johannsen et al., 2003). Moreover, longitudinal evidence indicates that regular physical activity during childhood and adolescence contributes positively to bone development and musculoskeletal health (Bland et al., 2020; Cronholm et al., 2020; Devulapalli, 2025). Therefore, the superior fitness profile observed in the handball group is consistent with the greater exposure to mechanical loading that may contribute to the skeletal adaptations identified in this study.

The most consistent skeletal finding of the present study was the greater BMD observed in handball players at follow‐up. Although the handball group was taller and heavier than controls, lean mass was comparable between groups at baseline, and its longitudinal increase did not differ significantly between groups. Therefore, the greater skeletal mineralization observed in the trained group cannot be explained simply by a greater increase in lean mass over time. Nevertheless, regression analyses showed that individual increases in lean mass were positively associated with changes in BMD and BMC, supporting the important contribution of muscle development to bone mineral acquisition during growth (Deng et al., 2021; López‐Peralta et al., 2022). These associations should, however, be interpreted as relationships between concurrent changes in muscle and bone rather than evidence of a direct mediating effect of lean mass.

After adjustment for baseline BMD and relevant growth‐related covariates, including height growth velocity, height, lean mass, and dietary calcium intake, handball players exhibited higher BMD at the whole body, lumbar spine, lower limbs, femoral neck, total hip, and selected upper‐limb sites. Furthermore, the greater relative increases in whole‐body and lumbar‐spine BMD observed in the handball group support the interpretation that these differences were not solely attributable to baseline skeletal characteristics or growth‐related factors. The BMC findings were broadly consistent with this pattern. Although the handball group displayed higher BMC values at several sites at baseline, adjusted analyses confirmed greater BMC at the lumbar spine, left leg, bilateral total hip, and right one‐third radius at follow‐up. In addition, whole‐body and lumbar‐spine BMC gains were greater in handball players than controls. Because BMC reflects the absolute amount of mineral contained within bone, the combination of greater BMD and BMC suggests enhanced skeletal mineral acquisition rather than differences attributable solely to body size.

The regional distribution of these skeletal adaptations was particularly evident at weight‐bearing sites. Greater BMD values were observed at both legs, the left femoral neck, and bilateral total hip regions, while BMC differences were also evident at the lower limbs, hip, and selected radial sites. This pattern is biologically plausible considering the mechanical characteristics of handball, which involve repeated jumping, landing, sprinting, rapid changes of direction, and multidirectional loading. Bone tissue is highly responsive to dynamic and impact loading during growth, and previous intervention and longitudinal studies have shown that high‐impact activities can increase bone mineral accrual at the total body, hip, and lower‐limb sites in children and adolescents (Bailey et al., 1999; Gustavsson et al., 2003; Johannsen et al., 2003; Vicente‐Rodríguez, 2006). Specific evidence from prepubescent and peripubertal boys is also consistent with the present findings. In a study of prepubescent handball players aged 9–12 years, Missawi et al. (2016) reported greater BMD at the femoral neck and total hip, as well as higher BMC at the lower limbs and total hip, compared with controls. Similarly, Zouch et al. (2014), in boys aged 10–13 years who were prepubescent at baseline, reported greater BMC at the whole body and weight‐bearing sites in soccer players compared with controls. These findings provide evidence of site‐specific bone adaptations to impact sports during childhood and the peripubertal period.

Interestingly, adaptations were also observed at selected non‐weight‐bearing sites, including the left arm, right ultradistal radius, and right whole radius. Although these regions are not exposed to the same body weight loading as the lower limbs, handball involves repeated throwing, catching, blocking, and other upper‐limb movements that may generate sport‐specific mechanical stimuli. Similar site‐specific skeletal responses have been described in unilateral upper‐limb sports across different developmental stages. In prepubertal male baseball players (8–11 years), throwing was associated with greater gains in humeral cortical bone mass and structural properties over a 12‐month period (Weatherholt & Warden, 2018). In contrast, among young adult male racquet‐sport athletes (22.46 ± 2.41 years), greater BMD was observed in the dominant compared with the non‐dominant upper limb (Ünver et al., 2021). These findings suggest that regional skeletal adaptations to unilateral mechanical loading may occur across different stages of development. Thus, the skeletal response to handball may not be restricted to weight‐bearing regions but may also reflect the multidirectional and sport‐specific nature of the mechanical stimuli generated during training and play.

In contrast to BMD and BMC, changes in BA were less pronounced. Although BA increased over time in both groups, no significant between‐group differences were observed for whole‐body or lumbar‐spine BA after adjustment for baseline values and covariates. Linear mixed‐effects models identified several nominal Group × Time interactions, but none remained significant after FDR correction. Some adjusted differences were detected at selected upper‐limb sites; however, these findings should be interpreted cautiously because they were not consistently supported by longitudinal analyses after correction for multiple comparisons.

The limited effect on BA may reflect the relatively short follow‐up period and the complexity of skeletal growth. Bone development involves partially independent processes, including longitudinal growth, periosteal expansion, cortical modeling, and mineral accumulation (Rauch, 2005). During childhood and early adolescence, physiological growth itself produces substantial increases in bone dimensions, potentially limiting the detection of additional exercise‐related effects on bone area over 1 year (Baxter‐Jones et al., 2003). Conversely, mechanical loading may induce detectable changes in mineral acquisition before measurable alterations in bone geometry, consistent with the mechanostat theory of skeletal adaptation (Rauch & Schoenau, 2001; Schoenau, 2005). The present findings, therefore, suggest that the skeletal response to regular handball participation during this period may be more evident in mineralization than in bone area.

The regression analyses provide additional insight into the relationship between muscular and skeletal development. Greater increases in lean mass were positively associated with BMD gains at the whole body, bilateral femoral necks, and bilateral total hip sites after adjustment for age, height, and calcium intake. These findings support previous evidence identifying lean mass as an important determinant of bone mineral acquisition during growth (Deng et al., 2021; López‐Peralta et al., 2022). The observed associations may reflect the influence of muscle‐generated forces on bone and the functional interaction between muscle and skeletal development described by the muscle–bone unit concept (Rauch & Schoenau, 2001; Schoenau, 2005). Associations with BMC were more localized, whereas associations with BA were limited to whole‐radius regions, further suggesting that changes in lean mass may be more closely related to skeletal mineral acquisition than to generalized changes in bone geometry.

Overall, the present findings indicate that regular handball participation during growth is associated with favorable skeletal characteristics and longitudinal changes, particularly in bone mineral accrual at mechanically loaded sites. The combined pattern of BMD and BMC findings, together with the limited differences in BA, suggests that the skeletal response was characterized primarily by enhanced mineral acquisition rather than by greater changes in bone size. The repeated impact loading, sport‐specific movements, and muscular development associated with handball may contribute to the mechanical stimuli underlying skeletal adaptation during this critical period of bone development (Cronholm et al., 2020; Johannsen et al., 2003).

These findings should nevertheless be interpreted cautiously. The regression analyses demonstrate associations but cannot establish mediation or causal pathways. In addition, baseline differences between groups and the non‐random allocation to training limit causal interpretation. The dietary assessment provides additional information on potential nutritional factors, including energy, protein, carbohydrate, fat, calcium, and vitamin D intake, but the observational design also precludes definitive conclusions regarding the independent contribution of handball participation. Nevertheless, the consistent pattern of greater BMD and BMC responses, particularly at weight‐bearing sites, supports the potential value of multidirectional and impact‐based activities for promoting bone development and optimizing peak bone mass acquisition during growth (Bailey et al., 1999; Baxter‐Jones et al., 2003; Karlsson & Rosengren, 2020).

A strength of the present study is the simultaneous assessment of BMD, BMC, and BA across multiple skeletal regions, providing a more comprehensive characterization of skeletal adaptation than BMD alone. Moreover, the use of adjusted ANCOVA models and linear mixed‐effects models strengthened the evaluation of between‐group differences and longitudinal changes.

Several limitations should be considered when interpreting the present findings. First, the non‐randomized design and baseline differences between groups limit the ability to establish a causal relationship between handball participation and changes in bone properties. The handball players had already participated in regular handball training for approximately 2 years before baseline and presented greater body mass, lean mass, and physical fitness than the controls. Consequently, the higher baseline BMD, BMC, and BA observed in the handball group may partly reflect the cumulative effects of previous physical activity and differences in body size, body composition, and physical fitness. Although our statistical models accounted for baseline bone values, height, growth velocity, lean mass, and dietary calcium intake, residual confounding due to previous training exposure and other unmeasured factors cannot be excluded. Thus, the changes observed during the 12‐month follow‐up should not be interpreted as being exclusively attributable to handball participation during this period. Rather, they may reflect the combined effects of previous and ongoing training, individual growth and maturation, and other characteristics distinguishing young athletes from non‐athletes. Therefore, site‐specific differences in BMD changes should be interpreted cautiously in light of these potential confounding factors. Future prospective studies beginning before the initiation of structured handball training are warranted to better determine the specific contribution of handball to skeletal development during the prepubertal period.

The relatively modest sample size and the evaluation of multiple skeletal sites may have reduced statistical power and increased the risk of false‐positive findings. Although FDR correction was applied, some nominal group × time interactions did not remain significant and should therefore be considered exploratory. Finally, the one‐year follow‐up period may have been insufficient to detect clear differences in bone geometry, which may require longer exposure to mechanical loading to become distinguishable from normal growth‐related changes.

## 5 CONCLUSION

Overall, the present findings indicate that regular handball participation during growth is associated with greater BMD and BMC accrual, particularly at weight‐bearing sites. In contrast, changes in BA were limited and did not show robust longitudinal differences after correction for multiple comparisons. The positive associations between lean mass gains and BMD improvements at clinically relevant regions, including the hip and femoral neck, further highlight the role of the muscle–bone interaction in skeletal development. These findings suggest that handball may represent a promising form of osteogenic physical activity during growth, with its skeletal effects being more evident for mineral acquisition than for generalized increases in bone size.

## AUTHOR CONTRIBUTIONS

**Kawther Missawi:** Conceptualization; formal analysis; investigation; methodology. **Maha Gasmi:** Conceptualization; formal analysis; methodology; resources. **Antonella Muscella:** Conceptualization; data curation; investigation; supervision. **Tahir Volkan Aslan:** Investigation; methodology. **Özgür Eken:** Investigation; methodology; project administration. **Monira I. Aldhahi:** Investigation; methodology; resources. **Mohamed Zouch:** Investigation; methodology; resources; supervision.

## FUNDING INFORMATION

The authors have nothing to report.

## CONFLICT OF INTEREST STATEMENT

The authors declare no conflicts of interest.

## ETHICS STATEMENT

All procedures were approved by the Ethics Committee of Farhat Hached Hospital, Sousse, Tunisia (LR19ES10), and conducted in accordance with the Declaration of Helsinki.

## Supporting information


**Table S1.** Linear mixed‐effects model results for anthropometric data and calcium intake.
**Table S2.** Group × Time interaction for BMD across skeletal regions.
**Table S3.** Group × Time interaction for bone mineral content (BMC) across skeletal regions.
**Table S4.** Group × Time interaction for bone area (BA) across skeletal regions.

## Data Availability

The data that support the findings of this study are available from the corresponding author upon reasonable request.
