# Supplementary material for: Handball participation and bone mineral accrual during growth: A 1‐year longitudinal controlled study in young male athletes
Source: Physiol Rep. 2026 Oct 13;14(19):e71133. doi: 10.14814/phy2.71133 (PMC13662231; doi:10.14814/phy2.71133)
Supplement: Supplementary file 1 [file PHY2-14-e71133-s001.docx]

**Table S1.** Linear mixed-effects model results for anthropometric data and calcium intake

| **Variable** | **Effect** | **F (df1, df2)** | **p** | **η²p** | **95% CI** |
| --- | --- | --- | --- | --- | --- |
| Height (m) | Group | 46.96 (1,105) | < 0.001 | 0.309 | 0.043 to 0.094 |
|  | Time | 30.82 (1,105) | < 0.0001 | 0.227 | 0.037 to 0.051 |
|  | G × T | 0.42 (1,105) | 0.519 | 0.004 | −0.009 to 0.019 |
| Body mass (Kg) | Group | 12.82 (1,104.61) | < 0.001 | 0.109 | 2.44 to 11.33 |
|  | Time | 6.24 (1,104.39) | 0.014 | 0.056 | 2.97 to 5.19 |
|  | G × T | 0.18 (1,104.39) | 0.676 | 0.002 | −0.35 to 1.18 |
| BMI | Group | 3.82 (1,39.61) | 0.058 | 0.088 | 0.05 to 3.16 |
|  | Time | 0.02 (1,62.02) | 0.903 | 0.000 | -0.3 to 0.84 |
|  | G × T | 2.04 (1,70) | 0.157 | 0.028 | −0.11 to 0.69 |
| Total lean mass (g) | Group | 5.99 (1,43.35) | 0.018 | 0.121 | 345.95 to 4513.88 |
|  | Time | 66.45 (1,70) | < 0.001 | 0.655 | 1726.9 to 3695.61 |
|  | G × T | 1.53 (1,70) | 0.225 | 0.042 | −1040.90 to 235.22 |
| Total fat mass (g) | Group | 5.59 (1,37.28) | 0.023 | 0.130 | 773.88 to 6588.85 |
|  | Time | 6.77 (1,64.47) | 0.011 | 0.095 | 41.61 to 1585.05 |
|  | G × T | 0.09 (1,70) | 0.769 | 0.001 | −458.42 to 620.56 |
| Calcium intake | Group | 0.30 (1,83.78) | 0.583 | 0.004 | -29.80 to 52.24 |
|  | Time | 2.64 (1,38.68) | 0.113 | 0.064 | -10.15 to 105.59 |
|  | G × T | 0.45 (1,70) | 0.503 | 0.006 | −35.06 to 17.14 |
| 20mSRT (distance) (m) | Group | 859.73 (1, 139) | <0.0001 | 0.861 | 312.55 to 359.51 |
|  | Time | 3.15 (1, 139) | 0.0780 | 0.022 | -42.25 to 78.20 |
|  | G × T | 11.38 (1, 139) | 0.0010 | 0.076 | 32.00 to 122.68 |

# **Table S2.** Group × Time interaction for BMD across skeletal regions

| Region | F(1, 138) | p-value | p (FDR) | η²p |
| --- | --- | --- | --- | --- |
| Whole Body | 5.728 | 0.017 | 0.091 | 0.0399 |
| Lumbar Spine | 5.866 | 0.015 | 0.091 | 0.0408 |
| Left Leg | 4.660 | 0.031 | 0.091 | 0.0327 |
| Right Leg | 2.085 | 0.149 | 0.281 | 0.0149 |
| Left Femoral Neck | 8.325 | 0.004 | 0.066 | 0.0569 |
| Right Femoral Neck | 0.527 | 0.468 | 0.568 | 0.0038 |
| Left Total Hip | 4.586 | 0.032 | 0.091 | 0.0322 |
| Right Total Hip | 5.086 | 0.024 | 0.091 | 0.0355 |
| Left Arm | 0.626 | 0.429 | 0.561 | 0.0045 |
| Right Arm | 0.011 | 0.916 | 0.916 | 0.0001 |
| Left 1/3 Radius | 0.208 | 0.648 | 0.689 | 0.0015 |
| Right 1/3 Radius | 1.764 | 0.184 | 0.313 | 0.0126 |
| Left Whole Radius | 0.755 | 0.385 | 0.545 | 0.0054 |
| Right Whole Radius | 3.907 | 0.048 | 0.102 | 0.0275 |
| Left Ultradistal Radius | 0.306 | 0.580 | 0.657 | 0.0022 |
| Right Ultradistal Radius | 4.127 | 0.042 | 0.102 | 0.0290 |

# **Table S3.** Group × Time interaction for bone mineral content (BMC) across skeletal regions

| Region | f (1, 138) | p-value | p (FDR) | ηp2 |
| --- | --- | --- | --- | --- |
| Head | 0.824 | 0.364 | 0.413 | 0.0059 |
| Whole Body | 3.736 | 0.053 | 0.208 | 0.0264 |
| Lumbar Spine | 5.790 | **0.016** | 0.137 | 0.0403 |
| Left Leg | 6.366 | **0.012** | 0.137 | 0.0441 |
| Right Leg | 3.505 | 0.061 | 0.208 | 0.0248 |
| Left Femoral Neck | 0.324 | 0.569 | 0.569 | 0.0023 |
| Right Femoral Neck | 1.619 | 0.203 | 0.295 | 0.0116 |
| Left Total Hip | 2.463 | 0.117 | 0.283 | 0.0175 |
| Right Total Hip | 1.009 | 0.315 | 0.383 | 0.0073 |
| Left Arm | 1.677 | 0.195 | 0.295 | 0.0120 |
| Right Arm | 4.621 | **0.032** | 0.179 | 0.0324 |
| Left 1/3 Radius | 0.406 | 0.524 | 0.557 | 0.0029 |
| Right 1/3 Radius | 1.585 | 0.208 | 0.295 | 0.0114 |
| Left Whole Radius | 1.479 | 0.224 | 0.295 | 0.0106 |
| Right Whole Radius | 1.467 | 0.226 | 0.295 | 0.0104 |
| Left Ultradistal Radius | 2.053 | 0.152 | 0.295 | 0.0147 |
| Right Ultradistal Radius | 3.005 | 0.083 | 0.235 | 0.0213 |

# **Table S4.** Group × Time interaction for bone area (BA) across skeletal regions

| Region | f (1, 138) | p-value | p (FDR) | ηp2 |
| --- | --- | --- | --- | --- |
| Region | 5.728 | 0.017 | 0.091 | 0.0399 |
| Whole Body | 5.866 | 0.015 | 0.091 | 0.0408 |
| Lumbar Spine | 4.660 | 0.031 | 0.091 | 0.0327 |
| Left Leg | 2.085 | 0.149 | 0.281 | 0.0149 |
| Right Leg | 8.325 | 0.004 | 0.066 | 0.0569 |
| Left Femoral Neck | 0.527 | 0.468 | 0.568 | 0.0038 |
| Right Femoral Neck | 4.586 | 0.032 | 0.091 | 0.0322 |
| Left Total Hip | 5.086 | 0.024 | 0.091 | 0.0355 |
| Right Total Hip | 0.626 | 0.429 | 0.561 | 0.0045 |
| Left Arm | 0.011 | 0.916 | 0.916 | 0.0001 |
| Right Arm | 0.208 | 0.648 | 0.689 | 0.0015 |
| Left 1/3 Radius | 1.764 | 0.184 | 0.313 | 0.0126 |
| Right 1/3 Radius | 0.755 | 0.385 | 0.545 | 0.0054 |
| Left Whole Radius | 3.907 | 0.048 | 0.102 | 0.0275 |
| Right Whole Radius | 0.306 | 0.580 | 0.657 | 0.0022 |
| Left Ultradistal Radius | 4.127 | 0.042 | 0.102 | 0.0290 |
